# Idiopathic Interstitial Pneumonias and COVID-19 Pneumonia: Review of the Main Radiological Features and Differential Diagnosis

**DOI:** 10.3390/tomography7030035

**Published:** 2021-08-31

**Authors:** Alessia Guarnera, Elena Santini, Pierfrancesco Podda

**Affiliations:** 1 Radiology Department, San Giovanni Addolorata Hospital, 00184 Rome, Italy; elensantini@tiscali.it (E.S.); pf.podda@gmail.com (P.P.); 2 Neuroradiology Unit, NESMOS Department, Sant’Andrea Hospital, La Sapienza University, 00189 Rome, Italy

**Keywords:** COVID-19, idiopathic interstitial pneumonia, HRCT, differential diagnosis, usual interstitial pneumonia, non-specific interstitial pneumonia, organizing pneumonia, acute respiratory distress syndrome

## Abstract

COVID-19 pneumonia represents a challenging health emergency, due to the disproportion between the high transmissibility, morbidity, and mortality of the virus and healthcare systems possibilities. Literature has mainly focused on COVID-19 pneumonia clinical-radiological diagnosis and therapy, and on the most common differential diagnoses, while few papers investigated rare COVID-19 pneumonia differential diagnoses or the overlapping of COVID-19 pneumonia on pre-existing lung pathologies. This article presents the main radiological characteristics of COVID-19 pneumonia and Idiopathic Interstitial Pneumonias (IIPs) to identify key radiological features for a differential diagnosis among IIPs, and between IIPs and COVID-19 pneumonia. COVID-19 pneumonia differential diagnosis with IIPs is challenging, since these entities may share common radiological findings as ground glass opacities, crazy paving patterns, and consolidations. Multidisciplinary discussion is crucial to reach a final and correct diagnosis. Radiologists have a pivotal role in identifying COVID-19 pneumonia patterns, reporting possible overlapping with long-lasting lung diseases, and suggesting potential differential diagnoses. An optimal evaluation of HRTC may help in containing the disease, in promoting better treatment for patients, and in providing an efficient allocation of human and economic resources.

## 1. Introduction

COVID-19 Pneumonia represents a challenging health emergency, due to the disproportion between the high transmissibility, morbidity, and mortality of the virus and healthcare systems possibilities: COVID-19 has tested countries’ healthcare systems resilience [1].

Literature on COVID-19 pneumonia clinical-radiological diagnosis and therapy is extremely fertile and has mainly focused on the most common differential diagnoses [2,3,4], while few papers investigated rarer differential diagnoses and of COVID-19 pneumonia overlapping on long-lasting lung pathologies.

Idiopathic Interstitial Pneumonias (IIPs) represent a vast category of lung diseases with kaleidoscopic radiological, clinical, and histopathological features as defined by the 2013 Idiopathic Interstitial Pneumonia Classification of the American Thoracic Society/European Respiratory Society [5].

The radiologist has a pivotal role in identifying COVID-19 pneumonia radiological features, reporting its possible overlapping with long-lasting lung diseases, and suggesting potential differential diagnoses. An optimal evaluation of HRTC may promote better treatment for patients and provide an efficient allocation of human and economic resources.

Our main goals are to provide the main radiological characteristics of COVID-19 pneumonia and Idiopathic Interstitial Pneumonias and to identify key radiological features for a differential diagnosis among IIPs and between IIPs and COVID-19 pneumonia.

## 2. COVID-19 Pneumonia

COVID-19 pneumonia is a viral pulmonary disease caused by severe acute respiratory syndrome coronavirus 2 (SARS Cov2) with high interpersonal transmission through virus inhalation [5,6]. Anamnesis of close contact with affected patients and peculiar signs and symptoms as anosmia and ageusia are key elements for the diagnosis, confirmed by Real Time-Polymerase Chain Reaction (RT-PCR) swab, which may take up to 24 h for a final diagnosis [7,8,9]. Unfortunately, patients may not always report close contact and may present generic symptoms as a mild fever, cough, or show an atypical presentation with diarrhea or be completely asymptomatic [7,8]. In case of unavailability of swabs, waiting for swab results, and in patients presenting with atypical symptoms, the radiologist has a crucial role in suggesting COVID-19 diagnosis.

Chest X-ray has been recognized as insensitive in early or mild COVID-19 infection and does not represent the radiological choice for an early diagnosis [10,11]. It is mainly used to monitor COVID-19 pneumonia evolution or to assess alternative diagnoses because it is time and cost-effective. Decontamination of the equipment is easier, quicker, and cheaper compared to a CT scanner [10,12]. HRTC is far more sensitive for the diagnosis or exclusion of early and mild forms of COVID-19 pneumonia, as well as for monitoring disease progression. These characteristics make HRCT the best technique for reaching an early diagnosis and isolating patients to contain the infection in a pandemic scenario [10].

**Figure 1:**
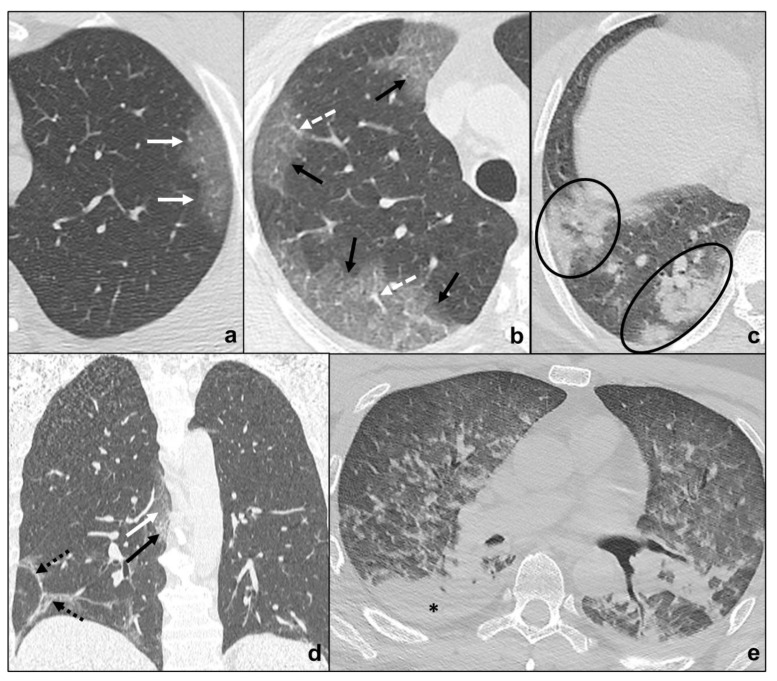
COVID-19 Pneumonia phases and possible evolutions. In (**a**), a subpleural ground-glass opacity (white arrow in (**a**)) indicating phase 1 is appreciable; in (**b**), extensive subpleural crazy paving pattern (black arrow in (**b**)) is preferrable to phase 2 and white dotted arrows indicate peripheral pulmonary vessel widening; in (**c**), consolidations (black circles in (**c**)) suggest phase 3; in (**d**), irregular consolidations coexisting (black dotted arrows in (**d**)) with ground glass opacities (black arrow in (**d**)) and crazy paving (black arrows in (**d**)) represent phase 4; in e, ARDS, a possible complication of COVID-19 pneumonia, presenting with asymmetric dependent consolidations (black asterisk in (**e**)) and widespread confluent ground glass opacities.

COVID-19 pneumonia has been divided into four stages, and the transition between two stages may carry overlapping findings [13] (Figure 1, Table 1):

1. Early phase/Stage 1 refers to days 0–4, and ground glass opacities represent the main radiological findings (Figure 1a);
2. Progressive phase/Stage 2 refers to days 5–8, and the hallmark is represented by crazy paving pattern (Figure 1b) coexisting with extensive ground glass opacities and initial consolidative foci;
3. Peak phase/Stage 3 is typical of days 9 to 13, and CT shows consolidations (Figure 1c), sometimes surrounded by a ground glass halo (halo sign);
4. Absorption phase/Stage 4 starts around day 14, and ground-glass areas together with linear consolidations are appreciable (Figure 1d).

Opacities are typically bilateral and subpleural with an apicobasal gradient of distribution. Additional radiological features are peripheral pulmonary vessel widening, while pleural effusions, pulmonary nodules, and mediastinal lymphadenopathy are rare findings [13,14,15].

Clinical conditions may worsen suddenly, and patients may show wheezing, hunger for air, tachypnea with reduced blood oxygen saturation. These features indicate COVID-19 pneumonia progression to ARDS (acute respiratory distress syndrome) [13,16]. ARDS radiologically presents on HRCT with patchy confluent areas of ground glass and dependent consolidations with a typical antero-posterior gradient [17]. Clinical and radiological monitoring are keys to the early identification and treatment of ARDS in COVID-19 pneumonia. HRCT is extremely sensitive to identify disease progression and complications, but it exposes patients to high radiation doses, is not cost-effective, and decontamination is far longer and more complex. On the other hand, chest X-ray is less sensitive, but cost and time effective with the advantage of easier decontamination [10,12]. For these reasons, chest X-rays may be used to monitor disease progression. The sudden onset of typical ARDS symptoms together with the identification of the abovementioned radiological features which do not match patients’ COVID-19 pneumonia phase should always be regarded as a possible sign of complications, such as ARDS.

## 3. Idiopathic Interstitial Pneumonias

The 2013 Idiopathic Interstitial Pneumonia (IIP) Classification of the American Thoracic Society/European Respiratory Society identified three categories [5] in relation to clinical, radiological, and pathological criteria: Major idiopathic interstitial pneumonias, rare idiopathic interstitial pneumonias, and unclassifiable idiopathic interstitial pneumonias.

**Figure 2:**
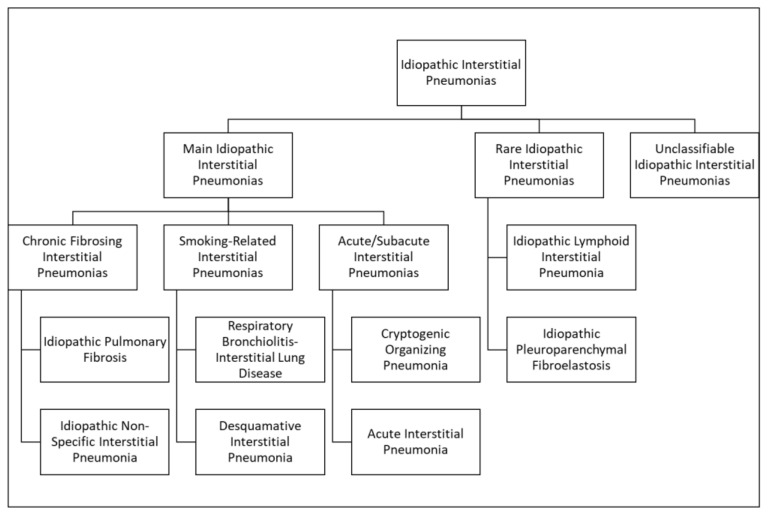
The chart schematically illustrates the 2013 Idiopathic Interstitial Pneumonia (IIP) Classification of the American Thoracic Society/European Respiratory Society [5].

Major IIPs include chronic fibrosing IIPs (NSIP/non-specific interstitial pneumonia and IPF/idiopathic pulmonary fibrosis), smoking-related IIPs (RB-ILD/respiratory bronchiolitis-associated interstitial lung disease, DIP/desquamative interstitial pneumonia), and acute/subacute IIPs (AIP/acute interstitial pneumonia and COP/cryptogenic organizing pneumonia). Rare IIPs include PPFE/pleuroparenchymal fibroelastosis and LIP/lymphoid interstitial pneumonia. Unclassifiable IIPs include pneumonias with no final diagnosis [5] (Figure 2; Table 2).

The gold standard for a final diagnosis is the multidisciplinary discussion, highlighting the importance of concordance among clinical, radiological, and pathological data [5]. In this context, the radiologist plays a pivotal role as the concordance of typical clinical and radiological presentation of IIPs may preserve patients from undergoing invasive procedures, such as pulmonary biopsy, i.e., certain UIP/IPF [18].

Therefore, what the radiologist should know are the main radiological features of each IIP and the differences among IIPs to reach a correct diagnosis. Chest X-rays are frequently normal or insensitive for a final diagnosis in the first stages, and high-resolution computed tomography (HRTC) represents the most sensitive and specific radiological technique to achieve a diagnosis [19].

Radiologists’ ability to reach a correct diagnosis highly rely on image quality. Raghu et al. [18] defined the optimal HRCT diagnostic protocol for diagnosing UIP, the most common among IIPs [19]. In this respect, the abovementioned protocol could be applied to all suspected IIPs to achieve a final diagnosis.

HRTC is preferentially acquired from volumetric scanning of the chest [20], with thinnest collimation [21], shortest rotation time, and highest pitch to guarantee high quality images with an optimal evaluation of subtle abnormalities, the possibility of post-processing (reformat, MIP/Maximum Intensity Projections, MinIP/Minimum Intensity Projection), and avoidance of movement artifacts [18]. It is recommended to acquire low-dose scans, avoiding ultralow-dose scans, with adjustment in tube dose and tube current in relation to patients’ sizes [22,23,24,25]. Three acquisitions are suggested: Supine position at sustained end-inspiration [26]; supine position at sustained end-expiration after a prolonged expiration, to detect air trapping [27]; and prone position at sustained end-inspiration to optimally analyze peripheral lung pathology without dependent lung atelectasis [27,28,29].

A systematic approach to HRTC may help to correctly diagnose or exclude IIPs. The pipeline entails evaluating image quality, the description of pathology features with standard terminology, and the analysis of pathology distribution within the lungs. The first differential diagnosis will be between a UIP pattern, and non-UIP patterns. Among non-UIP patterns, the radiologist should identify the presence of fibrotic features to further split the group into fibrotic IIPs and non-fibrotic IIPs. Ancillary features will help to identify the specific IIP [30].

### 3.1. Major Idiopathic Interstitial Pneumonias

Major idiopathic interstitial pneumonias are divided into: Chronic fibrosing IIPs, including idiopathic pulmonary fibrosis (IPF) and idiopathic nonspecific interstitial pneumonia (NSIP); smoking-related IIPs, including respiratory bronchiolitis-associated interstitial lung disease (RB-ILD) and desquamative interstitial pneumonia (DIP); acute/subacute IIPs, including cryptogenic organizing pneumonia (COP) and acute interstitial pneumonia (AIP) [5].

#### 3.1.1. Chronic Fibrosing IIPs

##### Idiopathic Pulmonary Fibrosis

Idiopathic pulmonary fibrosis is the most common IIP, presents with cough and dyspnea, and represents the clinical syndrome associated with a specific histological pattern called usual interstitial pneumonia (UIP) [5,30]. UIP is not pathognomonic of IPF, but may be secondary to collagenopathies, hypersensitivity pneumonia, pneumotoxic drugs resulting in a better prognosis. If UIP is secondary to IPF, and therefore, no cause is known, the prognosis is poor compared to the other IIPs [19].

An official ATS/ERS/JRS/ALAT clinical practice guideline providing clinical and radiological recommendations for diagnosing IPF was published in 2018. In particular, the guideline: Defined IPF diagnostic criteria; endorsed the importance of a detailed patient’s anamnesis, including medication use and environmental exposure; reaffirmed the pivotal role of the multidisciplinary discussion; gave indications on HRTC protocol and HRTC patterns; provided indications for serological testing, cellular analysis of BAL fluid and SLB/TBBx and criobiopsy [18]. Diagnosis of IPF requires excluding known causes of ILD; UIP pattern; combination of HRTC and histopathological pattern if lung biopsy is suggested [18]. UIP is divided into four patterns in relation to HRTC findings: UIP; probable UIP; indeterminate for UIP; alternative diagnosis [18].

**Figure 3:**
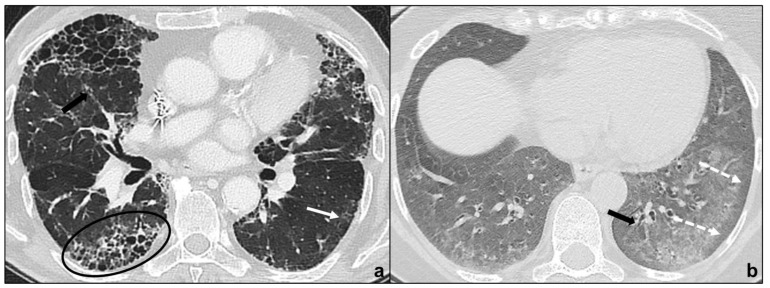
Major IIPs: Chronic Fibrosing IIPs. In (**a**), the UIP pattern is characterized by traction bronchiectases (black arrow in (**a**)), irregular reticular opacities, basal and subpleural macrocystic honeycombing (black oval in (**a**)), and non-prevalent ground-glass opacities (back arrow in (**a**)); in (**b**), the NSIP pattern is characterized by the bilateral, basal and diffuse ground glass with typical subpleural parenchymal preservation (white dotted arrows in (**b**)), coexisting with irregular reticular opacities and traction bronchiectasis (black arrow in (**b**)).

UIP is characterized by macrocystic honeycombing as a specific marker, architectural distortion with traction bronchiectases, irregular, reticular opacities, and decreased lung volumes [31]. Apicobasal gradient, heterogeneous involvement, and subpleural distribution are typical features [18]. Ground glass opacities are present, especially during disease exacerbation, but these opacities are limited in extent [19], and often accompanied by superimposed reticular patterns. There is a clear demarcation between healthy pulmonary parenchyma and UIP affected pulmonary parenchyma [5,32]. Occasionally, millimetric nodular calcifications may be appreciated [33]. Mediastinal lymphadenopathies [34] and coexisting emphysema may occur. Patients presenting with typical UIP features do not undergo lung biopsy and are diagnosed as IPF patients [18] (Figure 3a).

Probable UIP does not show radiological honeycombing, but the coexistence of peripheral traction bronchiectasis, subpleural and basal reticular abnormalities, and non-predominant ground-glass opacities; indeterminate for UIP is assigned to HRTCs demonstrating features of fibrosis without UIP/probable UIP criteria; alternative diagnosis is assigned to patterns suggesting a fibrotic lung disease different from IPF. In all previous cases, lung biopsy is required [18].

The radiologist has a crucial role in identifying a UIP pattern, in preventing the patient from undergoing a non-indicated biopsy, in describing UIP patterns requesting biopsies, or in suggesting alternative diagnoses.

##### Non-Specific Interstitial Pneumonia

NSIP presents with cough, dyspnea, and fatigue and may occur as an idiopathic condition or be secondary to underlying diseases, such as collagenopathies or to pneumotoxic drugs [19].

HRCT shows peripheral, basal, and symmetric ground glass opacities in the majority of patients, and irregular, reticular opacities and traction bronchiectases are extremely common findings [5,35,36]. The preservation of subpleural pulmonary tissue and the absence or sparse presence of microcystic honeycombing are typical and helpful in distinguishing NSIP from UIP [37]. Other key features to exclude UIP are the absence of an apicobasal gradient, finer reticular pattern and micronodules [19]. Consolidations are uncommon and suggest the co-presence of OP [5]. These radiological features suggest that a variable degree of fibrosis is appreciable in the majority of NSIP patients and justifies the inclusion in the chronic fibrosing IIPs [10] (Figure 3b).

#### 3.1.2. Smoking-Related IIPs

##### Respiratory Bronchiolitis-Associated Interstitial Lung Disease

**Figure 4:**
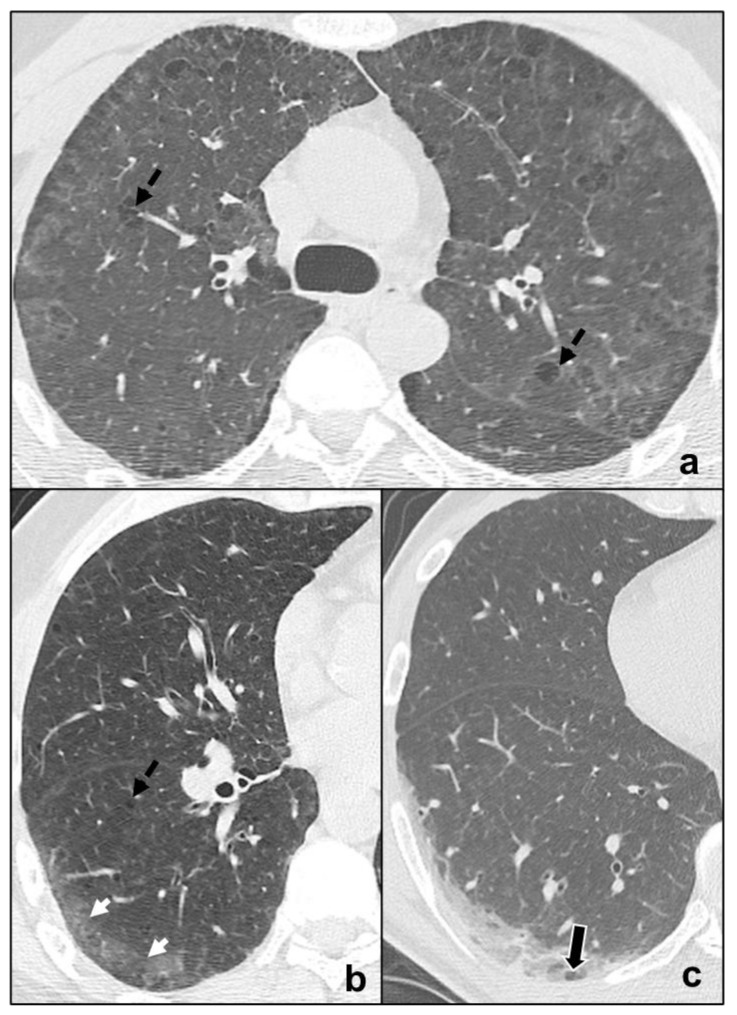
Major IIPs: Smoking-Related IIPs. In (**a**), RB-ILD is characterized by bilateral widespread ground glass opacities, centrilobular nodules, and centrilobular (black dotted arrows), and subpleural emphysema; in (**b**,**c**), DIP shows subpleural ground-glass areas (white arrow in (**b**)) associated with reticular, thin opacities and centrilobular (black dotted arrows) emphysema, while in (**c**), perivascular cysts are evident (white-bordered black arrow in (**c**)).

It is smoking-related IIP presenting with mild dyspnea and cough, diffusively affecting lungs or with upper pulmonary lobe predominance. Chest CT is characterized by centrilobular nodules, bronchial wall thickening, and ground glass opacities [38,39]. Frequently, coexisting centrilobular emphysema is described in relation to smoking habits [5] (Figure 4a).

##### Desquamative Interstitial Pneumonia

It is an IIP predominantly affecting smokers, but also documented in non-smokers and presenting with cough and dyspnea [19]. It is characterized by peripheral, basal ground glass opacities. Coexistence of linear or reticular opacities and perivascular cysts indicates fibrotic changes [5,40] (Figure 4b,c). Despite radiological differences with RB-ILD, differential diagnosis may be difficult with possible overlapping between the two pathologies.

RB-ILD and DIP should be distinguished from airspace enlargement with fibrosis (AEF), which represents an incidental radiological and/or pathological finding consisting in the coexistence between emphysema and interstitial fibrosis. AEF does not show clinically compared to pulmonary fibrosis and emphysema (CPFE), which represents the clinical manifestation of the same pattern [41,42,43].

#### 3.1.3. Acute/Subacute IIPs

##### Cryptogenic Organizing Pneumonia

**Figure 5:**
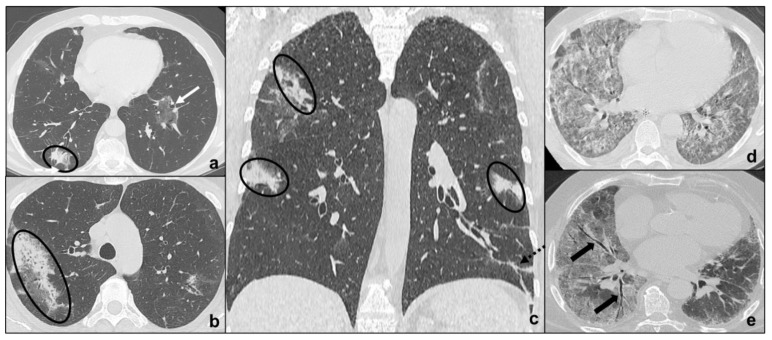
Major IIPs: Acute/Subacute IIPs. In (**a**–**c**), OP shows centro-parenchymal ground glass opacities (black arrow in (**a**)) coexisting with consolidations (black circles in (**a**–**c**)) and is characterized by typical subpleural parenchymal preservation (white dotted arrows in (**a**)), and irregular consolidations (black dotted arrow in (**c**)). In (**d**), exudative AIP is indicated by confluent, symmetric ground-glass opacities, and smooth septal thickening; while, in e, organizing AIP is characterized by architectural distortions, traction bronchiectases (black arrow in (**e**)), and microcystic honeycombing (white circle in (**e**)) coexisting with ground-glass opacities and consolidative foci.

OP may be idiopathic (COP) or secondary to collagenopathies, pneumotoxic drugs, cancer, or vasculitis and represents a stereotypical pulmonary reaction. OP presents with cough and dyspnea, which worsen during exacerbations and may be accompanied by fever [44]. In the initial stage, HRTC shows unilateral or bilateral patchy ground glass opacities evolving to consolidations with air bronchogram, surrounded by ground glass (reversed halo sign) in the late stages [45]. There is an apicobasal gradient with peribronchial or peripheral distribution [46]. In some cases, subpleural pulmonary tissue is spared [47,48]) similarly to NSIP and differently to UIP. OP opacities characteristically migrate, changing location and size; and increase over weeks of antibiotics [47]. Centrilobular nodules may be present and cavitate or coexist with irregular linear opacities [49,50] (Figure 5a–c). OP exacerbations may result in progressive interstitial fibrosis and represent a fibrosing variant of OP resembling NSIP [5]. Differential diagnosis between the two IIPs is suggested by the presence of consolidations in OP [5], although NSIP and OP may overlap in lung-dominant connective tissue disease [51], as they represent a stereotypical pulmonary response to external or internal agents.

##### Acute Interstitial Pneumonia

AIP patients show sudden onset of dyspnea with laboratory alterations and frequently need hospital admission. It is a pneumonia consisting of two main phases. During the early stage (exudative AIP), bilateral, symmetric, patchy ground glass opacities are predominant and may coexist with consolidations of the dependent segments of the lungs [52,53]. Then, in the late phase (organizing AIP), architectural distortions, traction bronchiectases, and honeycombing are common findings [54,55] (Figure 5d,e). Organizing AIP may progress to other IIP patterns as NSIP or to pulmonary fibrosis [56,57].

Exudative AIP should also be distinguished from acute exacerbation of IIPS, which is more common in IPF. Differential diagnosis is suggested by the overlapping of new bilateral patchy ground glass opacities and consolidations with a typical IPF pattern characterized by honeycombing, traction bronchiectases, and reticular opacities [58,59].

### 3.2. Rare Idiopathic Interstitial Pneumonias

Rare idiopathic interstitial pneumonias include lymphoid interstitial pneumonia and idiopathic pleuroparenchymal fibroelastosis. Rare histological patterns have been grouped under this category, but they do not represent distinct IIPs, and no specific radiological pattern has been identified [5].

#### 3.2.1. Lymphoid Interstitial Pneumonia

**Figure 6:**
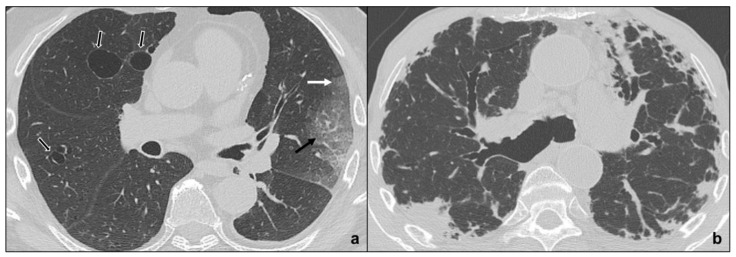
Rare IIPs. In (**a**), LIP is characterized by thin-walled perivascular cysts (white-bordered black arrows in (**a**)), and may coexist with subpleural ground glass opacities (white arrow in (**a**)) and crazy paving pattern (black arrow in (**a**)); in (**b**), PPFE is characterized by architectural distortions, traction bronchiectases, and subpleural fibrotic consolidations distributed bilaterally and predominantly at the upper fields.

It is a rare IIP presenting with cough and dyspnea and is not rarely associated with systemic immunodeficiency syndromes [5]. Chest CT demonstrates diffuse and basal ground glass opacities, centrilobular nodules, and septal thickening [35]. The hallmark of LIP is the presence of thin-walled perivascular cysts disseminated through the lung parenchyma with bilateral perivascular distribution [60] in contrast to UIP (Figure 6a).

#### 3.2.2. Idiopathic Pleuroparenchymal Fibroelastosis

PPFE is a rare IIP presenting with dyspnea and cough and associated with bone marrow transplantation, autoimmune disease, and genetic predisposition. PPFE is characterized by bilateral, subpleural fibrotic changes and pleural thickening, resulting in dense subpleural consolidations coexisting with architectural distortions, traction bronchiectasis, and hilar elevation leading to progressive upper lobe volume loss [5,32,61]. It shows upper field predominance, and pneumothorax is a common complication [5] (Figure 6b).

### 3.3. Unclassifiable Idiopathic Interstitial Pneumonias

Unclassifiable idiopathic interstitial pneumonias include pneumonias without a final diagnosis in relation to specific criteria: Absence of adequate clinical, radiological, and/or histological data; discordance among clinical, radiological, and pathological findings; and presence of various high-resolution CT patterns and/or pathological patterns suggesting idiopathic interstitial pneumonia. Major discordances in clinical, radiological, and pathological findings may be related to alteration of radiological and/or pathological findings secondary to therapy and to new or unusual entities [5].

## 4. Differential Diagnosis between COVID-19 Pneumonia and Idiopathic Interstitial Pneumonias

COVID-19 pneumonia and IIPs represent complex lung pathologies, and differential diagnosis is frequently challenging. Multidisciplinary discussion is pivotal to reach a final and correct diagnosis [5]. Radiological findings should always be correlated to patients’ anamnesis, clinical conditions, and laboratory. Chest HRCT should always juxtapose the patients’ previous HRCT exams.

Idiopathic Interstitial Pneumonias and COVID-19 Pneumonia are different entities, but share some radiological features, which may be analyzed and differentiated in relation to the different phases of COVID-19 pneumonia, as indicated in Table 3.

The approach we propose is to start by collecting a detailed patient’s anamnesis to identify possible close contacts with COVID-19 affected patients and to investigate the type and timing of symptoms. These last data are paramount since COVID-19 pneumonia is divided into four stages in which symptoms timing and radiological features are related [13]. The perfect match between typical symptoms and the correlated radiological picture may help to suggest COVID-19 diagnosis and guarantee patients’ isolation to avoid infection spread. A mismatch between patients’ anamnesis and Imaging should suggest a possible differential diagnosis. Comparison with previous HRCT scans is crucial to identify possible chronic or long-lasting radiological findings of IIPs. In particular, IIPs generally show the presence of typical and/or additional radiological findings which are uncommon or rare in COVID-19 pneumonia:

- tendency of the consolidations to migrate (OP) [47] (Figure 5a–c);
- preferential involvement of lobular periphery, with relative preservation of the lobular core, resulting in a “peri-lobular pattern” (OP) [5] (Figure 5a–c);
- ground-glass opacities during disease exacerbation, but limited in extent (IPF/UIP) [19] (Figure 3a);
- relative sparing of subpleural pulmonary tissue (NSIP, OP) [19] (Figure 3b and Figure 5a–c);
- predominance at the upper fields (RB-ILD, LIP, PPFE) [38,60,61] (Figure 4a and Figure 6);
- apicobasal gradient and heterogeneous involvement of the lungs (IPF/UIP) [62] (Figure 3a);
- clear demarcation between healthy pulmonary parenchyma and affected pulmonary parenchyma (IPF/UIP) [5,32] (Figure 3a);
- coexistence of other radiological findings as centrilobular nodules (RB-ILD, OP) and thin-walled cysts (LIP) [35,38] (Figure 4a, Figure 5a–c and Figure 6a);
- presence of fibrosis that may be appreciated as parenchymal distortion, traction bronchiectases and/or honeycombing (IPF/UIP, NSIP, OP, late-stage AIP, PPFE) [5,18,54,61] (Figure 3, Figure 5a–c,e and Figure 6b);
- pleural thickening (PPFE) [5,32,61] (Figure 6b);
- pleural effusions (exudative AIP, OP) [5] (Figure 5a–d).

Despite the abovementioned radiological differences between OP and COVID-19 pneumonia, OP has been speculated to be characteristic of “absorption stage” of COVID-19 pneumonia as a universal response to lung injury [2].

Finally, the differential diagnosis between AIP and ARDS may be extremely difficult, since clinical presentation may be similar. Some radiological findings differ between the two lung diseases, since AIP tends to present a more symmetric distribution with lower lung predominance compared to ARDS [30] in patients without a known cause and who did not undergo typical COVID-19 pneumonia phases.

## 5. Conclusions

COVID-19 pneumonia differential diagnosis with IIPs is challenging, since these entities may share common radiological findings as ground glass opacities, crazy paving patterns, and consolidations. Multidisciplinary discussion is crucial to reach a final and correct diagnosis. The radiologist should always collect a detailed anamnesis from correlating symptom types and timing to a COVID-19 pneumonia radiological features in relation to disease stage. The perfect clinical-radiological match may suggest COVID-19 diagnosis. A mismatch between patients’ anamnesis/clinical symptoms and Imaging should suggest a possible differential diagnosis. Comparison with previous HRCT scans is crucial to identify possible chronic or long-lasting radiological findings of IIPs. In particular, IIPs generally show the presence of typical and/or additional radiological findings (i.e., thin-walled cysts in LIP), which are uncommon or rare in COVID-19 pneumonia. An optimal evaluation of HRTC may help in containing the disease, in promoting better treatment for patients, and in providing an efficient allocation of human and economic resources.

## Figures and Tables

**Table 1:** COVID-19 pneumonia main radiological findings, additional features, and spatial distribution in relation to the four phases defined by Pan F. [13].

Stage	Phase	Timing (Days)	Predominant Radiological Findings	Additional Findings	Spatial Distribution of Radiological Findings
**1**	**Early**	0–4	ground glass opacities	peripheral vessel widening halo sign atoll sign or reversed halo sign overlapping of radiological findings in different phases rarity of: lymphadenopathies, pleural effusions, pulmonary nodules	bilateral peripheral/subpleural centro-parenchymal (atypical) lower lobes prevalence
**2**	**Progressive**	5–8	crazy paving pattern, ground glass opacities and small consolidations		
**3**	**Peak**	9–13	consolidative foci		
**4**	**Absorption**	≥14	ground-glass opacities and linear consolidation		

The table was reproduced from Guarnera A, Podda P, Santini E, Paolantonio P, Laghi A. “Differential diagnoses of COVID-19 pneumonia: The current challenge for the radiologist-a pictorial essay”, Insights Imaging. 2021 Mar 11;12 (1):34, under the terms and conditions of the Creative Commons Attribution (CC BY) license (http://creativecommons.org/licenses/by/4.0/ accessed on date 10 June 2021).

**Table 2:** The table illustrates typical CT features and patterns of distribution of the different IIPs.

Idiopathic Interstitial Pneumonias			Typical CT Features	Pattern of Distribution
**Main Idiopathic Interstitial Pneumonias**	**Chronic Fibrosing Interstitial Pneumonias**	Idiopathic Pulmonary Fibrosis	macrocystic honeycombing, architectural distortion, traction bronchiectases, irregular reticular opacities, decreased lung volumes, non-predominant ground glass opacities	apicobasal gradient, heterogeneous involvement, subpleural location
		Non Specific Interstitial Pneumonia	no apicobasal gradient, basal predominance, homogeneous involvement, symmetric, peripheral, subpleural sparing	ground glass, irregular reticular opacities, traction bronchiectases
	**Smoking-Related Interstitial Pneumonias**	Respiratory Bronchiolitis-Associated Interstitial Lung Disease	centrilobular nodules, bronchial wall thickening, ground glass opacities, centrilobular emphysema	diffuse or upper lobes predominance, centrilobular
		Desquamative Interstitial Pneumonia	ground glass, linear or reticular opacities, rare perivascular cysts	apicobasal gradient, peripheral
	**Acute/Subacute Interstitial Pneumonias**	Cryptogenic Organizing Pneumonia	ground glass, consolidations, rare centrilobular nodules and reticular opacities	apicobasal gradient, unilateral or bilateral, patchy distribution, peribronchial or peripheral, migration tendency, possible subpleural sparing
		Acute Interstitial Pneumonia	exudative: patchy ground glass, consolidation; organizing: architectural distortions, traction bronchiectases, honeycombing	bilateral, symmetric, dependent lung segments, basal
**Rare Idiopathic Interstitial Pneumonias**		Lymphoid Interstitial Pneumonia	perivascular thin-walled cysts, ground glass, centrilobular nodules, septal thickening	bilateral, basal, or diffuse
		Idiopathic Pleuroparenchymal Fibroelastosis	fibrotic changes, pleural thickening, consolidations, architectural distortions, traction bronchiectasis, hilar elevation, progressive upper lobe volume loss	bilateral, upper field predominance, subpleural

**Table 3:** The different IIPs generally share at least one radiological feature among ground-glass, crazy paving, and consolidations, with COVID-19 pneumonia, though often with different timing.

Idiopathic Interstitial Pneumonias			Ground Glass	Crazy Paving	Consolidations
**Main Idiopathic Interstitial Pneumonias**	**Chronic Fibrosing Interstitial Pneumonias**	Idiopathic Pulmonary Fibrosis	C (non predominant pattern, exacerbation)	R	R
		Non-Specific Interstitial Pneumonia	C	OD/A	R
	**Smoking-Related Interstitial Pneumonias**	Respiratory Bronchiolitis-Associated Interstitial Lung Disease	C	R	OD/A
		Desquamative Interstitial Pneumonia	C	OD/A	OD/A
	**Acute/Subacute Interstitial Pneumonias**	Cryptogenic Organizing Pneumonia	C (initial exacerbation)	OD/A	C (late)
		Acute Interstitial Pneumonia	C (early)	C	C (early)
**Rare Idiopathic Interstitial Pneumonias**		Lymphoid Interstitial Pneumonia	C	C/R	R
		Idiopathic Pleuroparenchymal Fibroelastosis	OD/A	OD/A	C

C, common; R, rare; OD/A, occasionally described/absent.

## Data Availability

The data are available from the corresponding author, A.G., upon reasonable request.

## Abbreviations

IIP, idiopathic interstitial pneumonia; IPF, idiopathic pulmonary fibrosis; UIP, usual interstitial pneumonia; NSIP, idiopathic non-specific interstitial pneumonia; RB-ILD, respiratory bronchiolitis-associated interstitial lung disease; DIP, desquamative interstitial pneumonia; AEF, airspace enlargement with fibrosis; CPFE, combined pulmonary fibrosis and emphysema; COP, cryptogenic organizing pneumonia; OP, organizing pneumonia; AIP, acute interstitial pneumonia; LIP, lymphoid interstitial pneumonia; PPFE, idiopathic pleuroparenchymal fibroelastosis; HRTC, high-resolution computed tomography; CT, computed tomography; SARS Cov2, severe acute respiratory syndrome coronavirus 2; RT-PCR, Real Time-Polymerase Chain Reaction.
